# Reactivation of Chromosomally Integrated Human Herpesvirus-6 by Telomeric Circle Formation

**DOI:** 10.1371/journal.pgen.1004033

**Published:** 2013-12-19

**Authors:** Bhupesh K. Prusty, George Krohne, Thomas Rudel

**Affiliations:** 1 Chair of Microbiology, Biocenter, University of Würzburg, Würzburg, Germany; 2 Electron Microscopy Core Facility, Biocenter, University of Würzburg, Würzburg, Germany; The University of North Carolina at Chapel Hill, United States of America

## Abstract

More than 95% of the human population is infected with human herpesvirus-6 (HHV-6) during early childhood and maintains latent HHV-6 genomes either in an extra-chromosomal form or as a chromosomally integrated HHV-6 (ciHHV-6). In addition, approximately 1% of humans are born with an inheritable form of ciHHV-6 integrated into the telomeres of chromosomes. Immunosuppression and stress conditions can reactivate latent HHV-6 replication, which is associated with clinical complications and even death. We have previously shown that *Chlamydia trachomatis* infection reactivates ciHHV-6 and induces the formation of extra-chromosomal viral DNA in ciHHV-6 cells. Here, we propose a model and provide experimental evidence for the mechanism of ciHHV-6 reactivation. Infection with *Chlamydia* induced a transient shortening of telomeric ends, which subsequently led to increased telomeric circle (t-circle) formation and incomplete reconstitution of circular viral genomes containing single viral direct repeat (DR). Correspondingly, short t-circles containing parts of the HHV-6 DR were detected in cells from individuals with genetically inherited ciHHV-6. Furthermore, telomere shortening induced in the absence of *Chlamydia* infection also caused circularization of ciHHV-6, supporting a t-circle based mechanism for ciHHV-6 reactivation.

## Author Summary

Human herpesviruses (HHVs) can reside in a lifelong non-infectious state displaying limited activity in their host and protected from immune responses. One possible way by which HHV-6 achieves this state is by integrating into the telomeric ends of human chromosomes, which are highly repetitive sequences that protect the ends of chromosomes from damage. Various stress conditions can reactivate latent HHV-6 thus increasing the severity of multiple human disorders. Recently, we have identified *Chlamydia* infection as a natural cause of latent HHV-6 reactivation. Here, we have sought to elucidate the molecular mechanism of HHV-6 reactivation. HHV-6 efficiently utilizes the well-organized telomere maintenance machinery of the host cell to exit from its inactive state and initiate replication to form new viral DNA. We provide experimental evidence that the shortening of telomeres, as a consequence of interference with telomere maintenance, triggers the release of the integrated virus from the chromosome. Our data provide a mechanistic basis to understand HHV-6 reactivation scenarios, which in light of the high prevalence of HHV-6 infection and the possibility of chromosomal integration of other common viruses like HHV-7 have important medical consequences for several million people worldwide.

## Introduction

Human herpesvirus 6 (HHV-6) is a ubiquitous pathogen with >90% seroprevalence in healthy adults. Although the process of viral latency is not completely understood, in some cases it is achieved by the integration of the viral genome into telomeric regions of host cell chromosomes (ciHHV-6) [1], and then subsequently vertically transmitted through the germ line [1]–[3]. Approximately 1% of the human population carries genetically inherited HHV-6. After becoming latent, HHV-6 persists in a dormant state with minimal viral transcription or translation in human host cells and without the production of infectious virions and any detectable clinical complications. However, under various physiological conditions, latent HHV-6 is reactivated and forms infectious viral particles (for further details see reviews [4]–[6]). Reactivated HHV-6 has been associated with various human diseases [7]–[10].

The ∼160 kb linear double-stranded genome of both species of HHV-6 (HHV-6A and -6B) is flanked by two distinct regions, ranging from 8 to 13 kb in length [11]–[13], called direct repeats (DR) at the left (DR_L_) and the right (DR_R_) ends of the genome, respectively. Multiple passages of HHV-6 in the laboratory have led to the shortening of DR due to the deletion of specific regions in DR_L_ and DR_R_ [11], [12]. Both the DR regions possess two well-defined stretches of telomeric repeats (T1 and T2). These repeat regions contain several copies of the sequence (TTAGGG)_n_, which are also found at the termini of linear eukaryotic chromosomes. The left end of each DR has short heterogeneous stretches of telomeric repeats (DR_L_-T1 and DR_R_-T1) whereas the right end of both the DRs has a single long stretch of homogeneous telomeric repeats (DR_L_-T2 and DR_R_-T2). Several other herpesviruses including Marek's disease virus (MDV) and HHV-7 have a similar genome organization containing multiple stretches of telomeric repeats at both ends. Although chromosomal integration of HHV-7 has not been identified so far, it has been suggested that homologous recombination between viral telomeric repeats and human telomeres mediates the integration of HHV-6 [14] and MDV [15] into the host cell genome. The presence of telomeric repeats within the viral genome may have dual functions, required for both the integration (to acquire latency) and its excision (to reactivate from latency) [14], [16]. However, the reactivation machinery and the exact mechanism for HHV-6 reactivation are currently unknown.

We have previously shown that replication of the ciHHV-6 genome is efficiently reactivated in blood cells of patients that are infected with *C. trachomatis* without the formation of viral particles [17]. In this study, we have used *Chlamydia* infection as a model to understand the mechanism of ciHHV-6 reactivation. Our results provide strong evidence for the existence of a t-circle based mechanism for the circularization of the integrated viral genome, which is possibly independent of the viral infectious cycle.

## Results

### A model for ciHHV-6 reactivation

**Figure 1:**
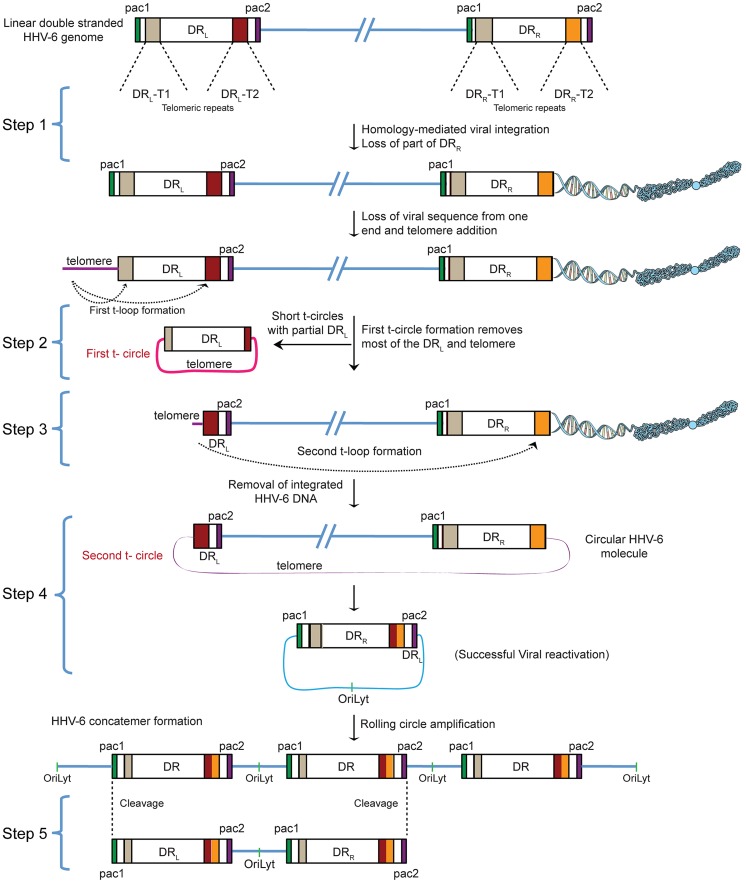
Proposed model for the reactivation of ciHHV-6. ciHHV-6 DNA has to be processed two times in order to form a t-circle containing a single DR, which can then undergo rolling circle amplification to form linear HHV-6 DNA. DR, direct repeats; DR_L_, left direct repeat; DR_R_, right direct repeat; T1, heterogeneous telomeric repeats; T2, homogeneous telomeric repeats; pac1 and pac2, packaging sequence 1 and 2; OriLyt, origin of lytic replication.

The reproducible and strong reactivation of ciHHV-6 replication by *Chlamydia* suggests that chlamydial infection triggers the exit of the integrated virus from the host genome and the subsequent formation of circular viral DNA, which functions as a template for rolling circle replication. Since previous work has demonstrated that HHV-6 contains telomere-like sequences within its genome and it integrates within the telomeres of eukaryotic chromosomes we investigated the role of these sequences in ciHHV-6 reactivation. We devised a hypothesis for the reactivation mechanism based on the following considerations: (1) since telomeres are dynamic DNA structures, they are subject to reorganization by the telomere maintenance machinery [18]. Therefore, the HHV-6 reactivation may be a consequence of the direct changes of telomeres at chromosomal ends. (2) The reactivated HHV-6 must form a circular DNA to allow rolling circle replication. (3) The circular HHV-6 DNA must maintain at least one complete and reconstituted DR with the packaging signal sequences pac1 and pac2. These sequences are required for the packaging and assembly of intact viral particles. (4) If the telomeric repeats mediate viral reactivation, excised extra-chromosomal HHV-6 should have variable telomeric-sequence lengths. Based on these considerations, we propose the model presented in Figure 1 as a basic mechanism for the formation of complete and reconstituted viral DNA.

HHV-6 integrates into telomeric regions of human chromosomes possibly by homologous recombination [2], [14] and in the process loses the distal end of the viral genome including the DR_R_- pac2 signal sequence essential for the packaging of viral DNA [2], [16] (Step 1; Figure 1). During the cell cycle, telomeric repeats can be added to the end of chromosomes by the cellular telomerase complex containing the RNA subunit hTR and the catalytic subunit, hTERT. We therefore propose that the overhanging end (step 1, DR_L_; Figure 1) of ciHHV-6 will progressively shorten during subsequent cell divisions until the host telomerase complex is able to bind to the exposed heterogeneous viral telomeric repeats and add telomeric repeats to stabilize the chromosomal ends. This process should lead to the loss of the pac1 sequence from the DR_L_ of the integrated viral genome (Figure 1). We hypothesize two ways by which the viral genome can subsequently be excised from the chromosome: (i) by homologous recombination within the integrated viral genome leading to circular viral DNA formation possibly mediated by the telomeric circle (t-circle) formation machinery of the host cell or (ii) by telomeric loop (t-loop) formation by branch migration and Holliday junction resolution (See reviews [18]–[20]). T -circles are duplex or single-stranded extra-chromosomal DNA circles formed from telomeric repeat sequences at the ends of human chromosomes and play a key role in maintenance of telomeres [20] whereas t-loops are loop like structures, frequently found at telomeric ends of chromosomes, which stabilize the telomere through the formation of the multiprotein shelterin complex [18]. T-loop formation is frequently observed in many different cell types; whereas single homologous recombination events during alternate lengthening of telomeres is suggested to form circular DNA molecules (t-circles) in the absence of telomerase activity [21], [22]. Since the extra-chromosomal HHV-6 DNA must be in a circular form for rolling circular replication, we considered both the t-circle and/or t-loop formation as possible mechanisms by which ciHHV-6 can form extra-chromosomal circular viral DNA.

There are two major possibilities for the successful reconstitution of the circular viral genome. A homologous recombination event between DR_L_-T1 and DR_R_-T1 would lead to the formation of a circular viral genome with a fixed length of DR-T2 and the presence of an incomplete DR_L_ in the human genome of reactivated cells. Alternatively, the left end of the DR_L_ could be first removed by a shorter t-circle formation process (Step 2, Figure 1), resulting in telomeric elongation of the chromosomal end from a homogenous telomeric repeat region of DR_L_ (DR_L_-T2) (Step 3, Figure 1). A second t-circle formation between DR_L_-T2 (Step 4, Figure 1) and DR_R_-T2 would result in the excision of the whole viral genome from the chromosome thereby generating a circular viral genome with a single reconstituted DR (Step 4, Figure 1). The circular viral genome can further undergo rolling circle amplification to form concatemeric viral DNA that can be cleaved to form linear double-stranded viral DNA molecules including two complete DR_L_ and DR_R_ sequences (Step 5, Figure 1).

### *Chlamydia* infection induces transient telomere shortening

**Figure 2:**
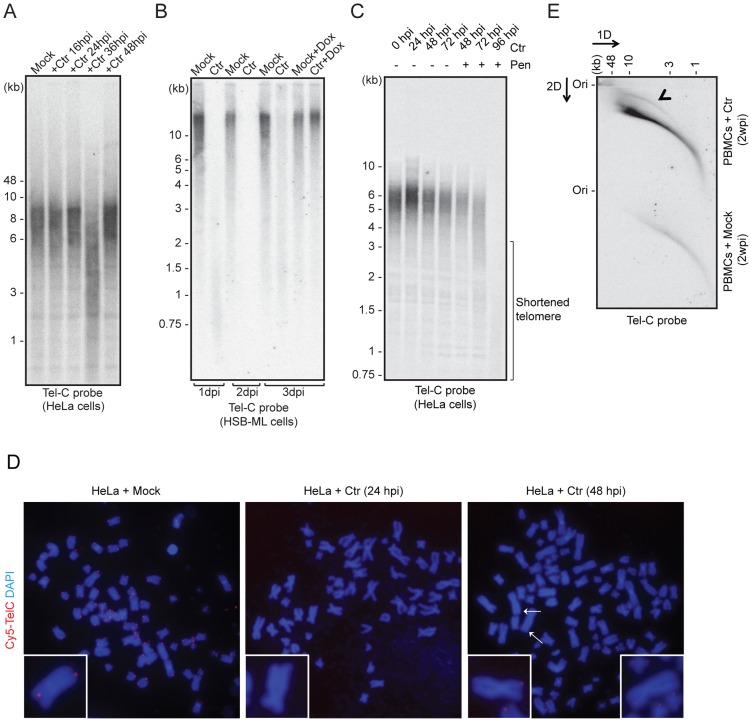
*C. trachomatis* (Ctr) infection induces telomere alterations. (**A**) HeLa (wild type) (**B**) HSB-ML cells were infected with *C. trachomatis* for different time intervals. Mock-infected or 1 µg/ml doxycycline (Dox)-treated cells were used as control. Total genomic DNA (10 µg) was digested with MspI and HhaI and telomeric sequences were detected by Southern hybridization using an end-labeled (CCCTAA)_4_ probe (Tel-C probe). The positions of molecular weight markers are shown on the left. Dox was added to cells wherever mentioned (+Dox) after 24 h post Ctr infection and the cells were grown for further time intervals. Mock, cells with no *Chlamydia* infection. (**C**) Penicillin (Pen)-induced *Chlamydia* persistence prevents telomere repairing. Where indicated, 100 U of penicillin was added 24 h post *Chlamydia* infection. (**D**) FISH analysis of metaphase chromosomes showing telomere shortening in HeLa cells infected with *Chlamydia*. Telomeres were hybridized with a Cy5-tagged telomeric C-rich (Tel-C) probe and are shown in the red channel; DNA was stained with 4′,6-diamidino-2-phenylindole (DAPI) and is shown in the blue channel. Arrows indicate missing telomeric staining from single sister chromatids. Representative individual chromosomes are enlarged to show telomere staining. (**E**) Detection of circular telomeric DNA in PBMCs after *Chlamydia* infection. Freshly isolated PBMCs from healthy individuals were either mock infected or with Ctr for 2 days. Subsequently, cells were cultured for 2 more weeks in presence of doxycycline. Genomic DNA from each sample was digested with HinfI and RsaI and was processed for 2D-DNA electrophoresis and subsequent Southern hybridization with Tel-C probe. Black arrowhead indicates circular telomeric DNA. hpi, hour post infection; dpi, days post infection; wpi, week post infection.

Reactivation of ciHHV-6 from human telomeres may be initiated by structural changes at the chromosomal ends. To analyze whether chlamydial infection has any impact on chromosomal ends, changes in telomere length were measured by telomere restriction fragment analysis during *C. trachomatis* infection. This assay determines telomere lengths by digesting DNA with frequently cutting restriction enzymes that do not cleave within telomeric sequences. Interestingly, we observed strong telomere shortening between 24–36 h of *Chlamydia* infection in different cell types including wild type HeLa (Figure 2A), HeLa229 (Figure S1A) and one of the ciHHV-6A cell lines (HSB-ML) (Figures 2B), which was followed by partial repair of telomeric ends. The presence of any nonspecific DNA degradation during *Chlamydia* infection was excluded by control hybridizations (Figure S1A). Termination of chlamydial infection by the addition of doxycycline, 24 h post infection, prevented telomere shortening indicating an active involvement of *Chlamydia* in this process (Figures 2B, S1A). Interestingly, persistent *Chlamydia*, a viable but non-productive form of *Chlamydia* induced by addition of penicillin, inhibited telomere repair (Figure 2C), suggesting that both telomere shortening and repair are actively induced by *Chlamydia* infection. The loss of telomeric sequences and subsequent defective telomere repair were also detected by fluorescent *in situ* hybridization (FISH) 48 h after *Chlamydia* infection (Figure 2D), where several single chromatids in *Chlamydia* infected cells showed weak or no telomeric signal (Figure 2D).

To test if HHV-6 reactivation involves t-circle formation after *Chlamydia* infection, we applied neutral-neutral 2D DNA electrophoresis. This method enables the discrimination between linear and circular DNA of the same size (Figure S1B) due to their differential migration in agarose gels [23]. This unusual migration behavior of DNA can be further resolved by either increasing the voltage or agarose concentration. Based on this principle, DNA is separated in two different dimensions during 2D DNA electrophoresis, first according to mass and then according to shape. The method has been previously extensively used to study the organization of telomeric DNA [23]–[28]. Interestingly, we detected increased t-circle formation in primary human PBMCs (Figure 2E) as well as in HeLa cells (Figure S1C) after *Chlamydia* infection. Thus, our data provide strong evidence for changes in telomere length and increased t-circle formation during *Chlamydia* infection, which can contribute to ciHHV-6 excision from human telomeres and subsequent reactivation.

### Reactivated HHV-6 genomes contain telomeric repeats of different length and exist in different conformations

**Figure 3:**
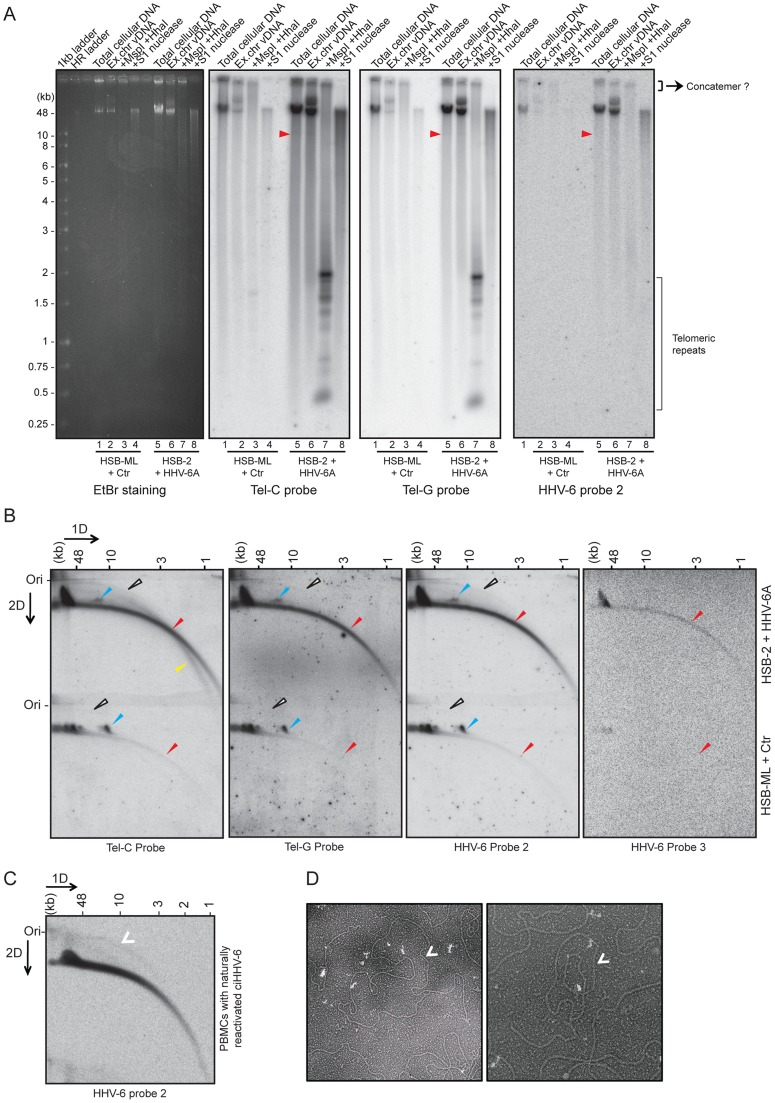
Extra-chromosomal HHV-6 DNA exists in different conformations and contains telomeric repeats of different sizes. (**A**) Purified viral DNA was either digested with MspI and HhaI or with S1 nuclease. All DNA samples were subjected to Southern hybridization using end-labeled (CCCTAA)_4_ probe (Tel-C probe), (TTAGGG)_4_ probe (Tel-G probe) or a HHV-6 DR specific probe (HHV-6 probe 2). HSB-ML+Ctr, *Chlamydia*-infected HSB-ML cells; HR ladder, high-range DNA ladder. The position of molecular weight markers is shown on the left. Smaller viral DNAs (possible short t-circles) are marked with red arrowhead. Possible concatemeric viral DNA are indicated. (**B**) Total genomic DNA from each cell type was processed for 2D-DNA electrophoresis and subsequent Southern hybridization with telomere-specific probes as well as with HHV-6 probes 2 and 3. Colored arrowheads mark different forms of viral DNA. White arrowheads: double-stranded circular HHV-6 DNA; Red arrowheads: double-stranded linear DNA; Blue arrowheads: short t-circles containing HHV-6 DR; Yellow arrowheads: single-stranded viral DNA (G-strand). DR_L_: left direct repeat. The positions of the size markers for the first-dimension gel electrophoresis are shown at the top, the origin of the second-dimension gel electrophoresis is indicated (Ori). (**C**) Detection of double-stranded full length circular HHV-6 DNA in PBMCs of ciHHV-6 patients with natural viral reactivation and with *in vitro* HHV-6 reactivation. Total genomic DNA from each cell type was processed for neutral-neutral 2D-DNA electrophoresis and subsequent Southern hybridization with HHV-6 probe 2. White arrowheads indicate double-stranded circular HHV-6 DNA. (**D**) Defined DNA fragments of ∼10 kb were gel eluted and prepared for EM by surface spreading. Shown is a reverse contrast image. Arrows mark circular t-circles of different sizes.

The presence of well-defined stretches of telomeric repeats within the viral DR could potentially lead to the formation of t-circles of variable sizes thereby generating viral DNA containing telomeric repeats of differing lengths. To investigate the excision of viral genomes as a consequence of t-circle formation, we investigated two different viral reactivation scenarios. We have frequently observed viral integration into host cell chromosomes during productive infection of HSB-2 cells. These cells productively infected with HHV-6A (Figure S2A) contained a fraction of the replicating viruses integrating into the telomeres (Figure S2B) and therefore could serve as source for virus replication and reactivation. Alternatively, we selected HSB-ML cells, which in contrast harbor ciHHV-6A and undergo viral reactivation and formation of extra-chromosomal viral DNA upon infection with *Chlamydia* (see Materials and Methods for details). To compare the length of tandem arrays of telomeric repeats, extra-chromosomal viral DNA was isolated from these cells using low melting DNA gel electrophoresis and then subjected to telomere restriction fragment analysis. In addition, S1 nuclease digests were used to monitor the amount of single-stranded nucleic acids in the isolated DNA. Differential lengths of telomeric repeats ranging from 0.25 to 2 kb in length were detected during productive HHV-6 infection in HSB-2 cells and in HSB-ML cells after virus reactivation (Figure 3A).

To check for the presence of multiple conformations of extra-chromosomal viral genomes, 2D DNA electrophoresis was performed with total DNA from HHV-6A-infected HSB-2 cells and ciHHV-6 HSB-ML cells reactivated by chlamydial infection. We detected circular HHV-6 DNA (marked with a white arrowhead in Figure 3B) in addition to the linear viral genome (marked with a red arrowhead) in HSB-2 cells, which co-hybridized with two different telomeric probes as well as with the HHV-6 specific probe (Figure 3B). However, HSB-ML cells contained very low amounts of circular DNA upon reactivation by *Chlamydia* infection. In addition, we observed a distinct band of viral DNA that was smaller in size in both samples from the different cell-lines (Figure 3B, blue arrow head), which may represent a shorter form of circular viral DNA. Full-length circular as well as linear double-stranded HHV-6 DNA was also detected in the total DNA isolated from primary PBMCs from one ciHHV-6 patient (Figure 3C) with ongoing natural viral reactivation at the time of blood sample collection. During further analysis, single-stranded circular HHV-6 DNA was detected in *Chlamydia* reactivated HSB-ML cells (marked with yellow arrowhead in Figure S2C), whereas this form was hardly detectable in HHV-6A infected HSB-2 cells (Figure S2C). Single-stranded circular viral DNA was clearly longer in size than the average telomeric circle in eukaryotic cells (Figure S2C) and was not present in control DNA samples (Figure S2D). S1 nuclease treatment digested both forms of circular DNA (Figure S2E) thereby confirming the presence of nicked circular double-stranded and/or single-stranded circular viral DNA in HHV-6A-infected cells.

To verify the presence of short t-circles in ciHHV-6A harboring HSB-ML cells, we gel purified DNA bands of approximately 10 kb in size (verified for presence of HHV-6 by southern hybridization) and performed TEM analysis. We observed mostly circular DNAs of varying sizes (Figure 3D) whereas control DNA preparations from uninfected HSB-2 cells (HHV-6A negative) did not contain any circular DNA. Thus, these results support our hypothesis that short t-circles carrying viral DR are formed in ciHHV-6 cells.

### Shortened telomeric ends at DR_L_-T2 of ciHHV-6 are generated by frequent t-circle formation

The absence of homogeneous stretches of telomeric repeats at DR_L_-T1 suggested that telomere addition and chromosomal end maintenance might begin from the DR_L_-T2 region. Therefore, we expected the formation of shorter t-circles between telomeric repeats added to DR_L_-T1 and DR_L_-T2 (Step 2, Figure 1). This process should generate a shorter DR_L_ without most of the DR_L_ ORFs. To test this hypothesis, we performed Southern hybridization analysis with total DNA from HSB-2 cells with both, chromosomally integrated HHV-6 and ongoing productive viral infection (Figures S2A, S2B) and from uninfected HSB-2 cells (Figure S3A). Re-hybridization of the same blot with 2 different HHV-6 probes complementary to the two ends of the DR_L_ as well as with a telomere specific probe confirmed the formation of short t-circles in these cells. DNA bands of the same length, detected by 2 different HHV-6 probes, could also originate from head-to-tail fused DNA concatemers. However, the length of head-to-tail fused concatemers should differ from those originating only from short t-circle formation (described in detail in Figures S3B, S3C), supporting the hypothesis that frequent t-circle formation removes parts of the viral DR_L_ leading to the generation of short circular DNA molecules containing viral DR.

**Figure 4:**
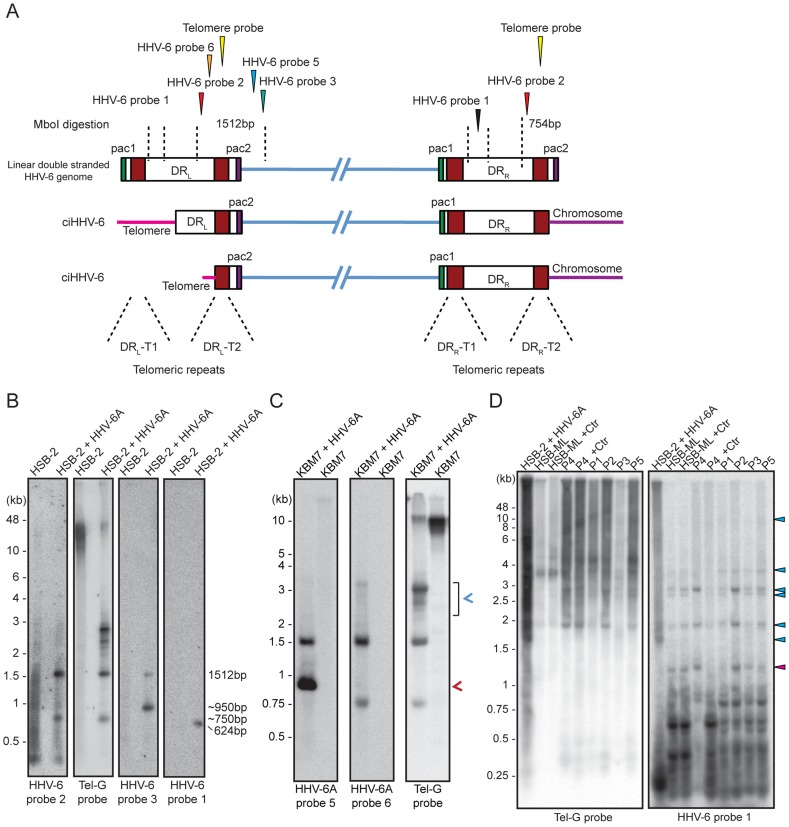
Detection of differentially processed ciHHV-6 ends. (**A**) Diagram of possible processing of the ciHHV-6 DR_L_-T2 end after viral integration. Approximate location of MboI digestion sites within and around viral DR are marked with dotted lines. The location of probes used to detect specific regions of viral DNA and their expected sizes are indicated. Different positions marked are based on HHV-6A U1102 genome (X83413.1). (**B**) Total genomic DNA from HSB-2 cells, with or without HHV-6A infection, were digested with MboI and processed for Southern hybridization. The membrane was stripped and re-probed sequentially with HHV-6 probe 2, Tel-G probe, HHV-6 probe 3 and finally with HHV-6 probe 1 (See table S1). (**C**) Similar experiment was performed in KBM-7 cells using HHV-6A probe 5, 6 and Tel-G probe. Chromosomal ends having DR_L_-T2 overhang is marked with red arrowhead. Short t-circles, which can be detected using Tel-G probe and HHV-6A probe 5 but not with HHV-6A probe 6 are marked with blue arrowhead. The positions of molecular weight markers are shown on the left. (**D**) Inverse PCR using forward and reverse primer directed out of the DR_L_ (see Figure S4B for orientation). Equal amount of the PCR products were subjected to Southern hybridization using probes against telomeric repeats (Tel-G probe). The membrane was rehybridized with a second probe against viral DR (HHV-6 probe 1). P1–P5 represents 5 different DNA samples from ciHHV-6 individuals. P4+Ctr represents PBMCs from P4 after 2 weeks of *Chlamydia* infection. The positions of molecular weight markers are shown on the left. The bands, which hybridize with both the probes, represent the extra-chromosomal short t-circles (blue arrow head). The band marked with red arrowhead represents short t-circles with extremely short telomeric repeats, which do not hybridize with telomeric probe.

T-circle formation between DR_L_-T1 and DR_L_-T2 (Step 2, Figure 1) should result in chromosomal ends having overhanging DR_L_-T2 (Step 3, Figure 1). To test this, we performed Southern hybridization analysis with total DNA from HHV-6 infected and uninfected HSB-2 cells and detected an approximately 950 bp fragment in infected HSB-2 cells with a probe hybridizing outside of the DR_L_ that was not detected by a DR-specific probe (Figures 4A, 4B). Interestingly, this band gave a poor telomeric hybridization signal, indicating the presence of an extremely short telomere at its left end. Similar restriction digestion and Southern hybridization experiment was carried out using total DNA from a haploid chronic myeloid cell line (KBM-7) [29], infected with HHV-6A, which allows productive virus infection (Figure S4A). Viral DNA ending at DR_L_-T2 as well as different sizes of short t-circles were detected in these cells (Figure 4C). Thus, the data suggest that part of the integrated HHV-6 DNA lacks DR_L_-T1 and contains a very short telomeric overhang starting at DR_L_-T2.

We detected distinct bands of short circular viral DNAs in HSB-2 cells with productive viral infection and in the *Chlamydia* infected HSB-ML cells (Figure 3B, marked with blue arrowhead). These short circular DNAs did not hybridize with a probe located in the U1 region, outside the viral DR, demonstrating that these short t-circles do not contain viral DNA outside the DR. The size of these bands correlate with the shorter DNA fragments from gel purified extra-chromosomal DNA (Figure 3A, marked with red arrowhead) indicating that short circular DNA molecules containing HHV-6 DR (Step 2, Figure 1) are frequently present in these cells and can be detected by various methods. Since ciHHV-6 blood DNA samples cannot be assessed for the presence of short t-circles by Southern analysis due to an insufficient DNA yield, we used inverse PCR to test for the presence of short t-circles in total DNA from freshly isolated ciHHV-6 PBMCs as well as some of the previously described cell lines carrying ciHHV-6 (detailed experimental approach is described in Figure S4). We amplified fragments of different sizes (Figures 4C), which were subsequently confirmed by southern hybridization using multiple probes and in part by sequencing to distinguish between short DR_L_-T1 and DR_L_-T2 containing t-circles (see Text S1). Variable lengths of telomeric repeats were detected in all the sequenced products, which did not show any major sequence differences within the viral DR. Our data indicate that shorter t-circle formation from ciHHV-6 DR_L_ is a frequent event since several different sizes of short circular DNA containing partial DR_L_ and its telomeric repeats were detected in all the cell types tested. Minor differences in the length of short t-circles were observed in the total DNA isolated before and after *Chlamydia*-mediated ciHHV-6 reactivation in HSB-ML cells and in one sample from ciHHV-6 PBMCs (P4) (Figure 4D). We detected several smaller amplification products using the HHV-6 probe 1 (Figure 4D), which was not detected by telomere probes. This results from the generation of incomplete PCR products due to difficulties in amplification of DNA from within highly GC-rich telomeric repeat regions. Thus, these results confirm our hypothesis that short extra-chromosomal t-circle formation is a frequent event and it is not altered by the subsequent reactivation of the viral DNA.

### Reactivated circular HHV-6 DNA contains a single DR

**Figure 5:**
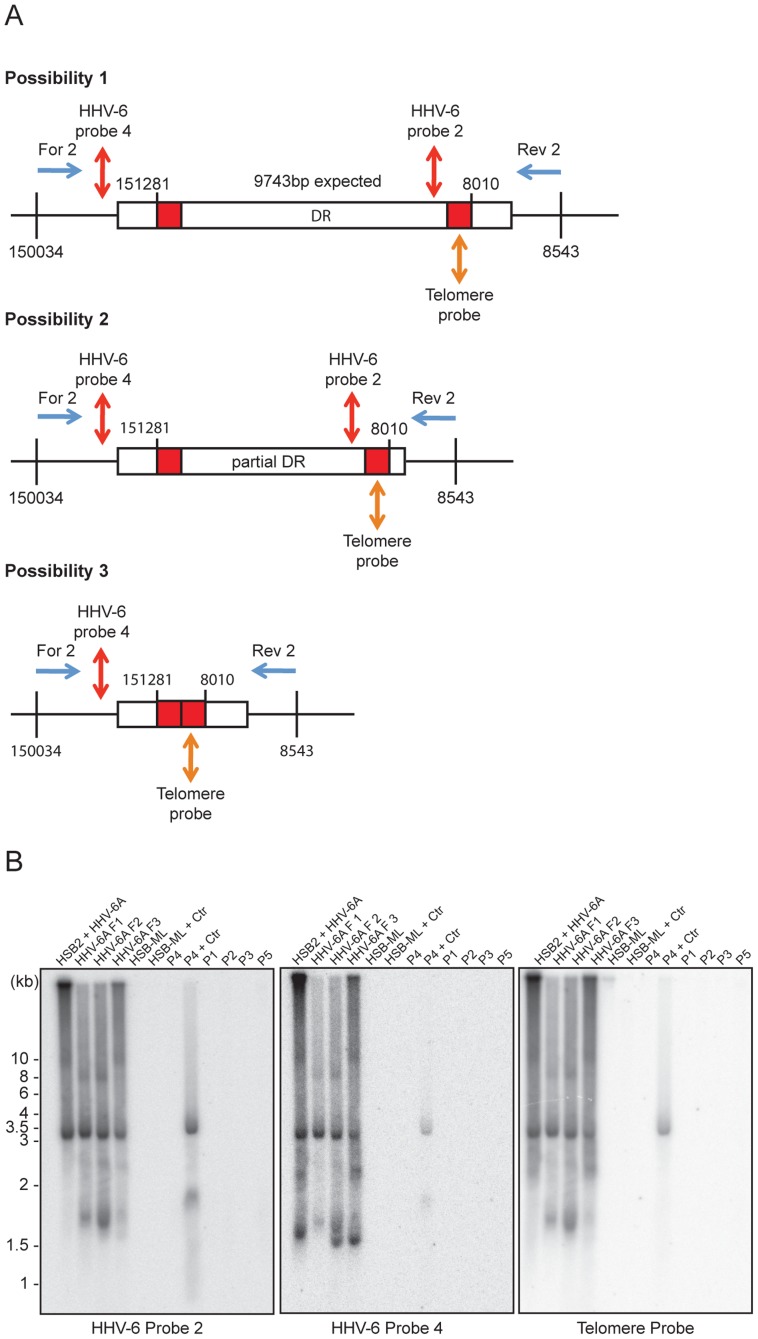
PCR amplification of circular HHV-6 genomes having a single direct repeat (DR). (**A**) Location of primers and southern hybridization probes to detect each possible combination of circular viral DNA (see table S1). Different positions marked are based on the HHV-6A U1102 genome (X83413.1). (**B**) Total DNA was used for PCR using a primer pair facing against each other but located outside the viral DR (see table S1). PCR products were used for Southern hybridization using HHV-6 probe 2. The membrane was re-hybridized with HHV-6 probe 4 and later on with telomere (Tel-G) probe. P1–P5 represents 5 different DNA samples from ciHHV-6 individuals. P4+Ctr represents PBMCs from P4 after 2 weeks of *Chlamydia* infection. The position of molecular weight markers run is shown on the left.

Validation of the final steps of the proposed model for HHV-6 reactivation required the detection of circular viral DNA with a single reconstituted DR (Step 4, Figure 1). We used inverse PCR with an inverse primer pair (table S1) located outside the DR (Figure 5A) to amplify circular viral DNA from several different cell types (Figures 5A, B). Once again, variable lengths of telomeric repeats were observed in extra-chromosomal HHV-6 DNA from HSB-2 cells (Figure 3A). Therefore, we size fractionated the full-length HHV-6A DNA from HSB-2 cells and used it separately for inverse PCR and subsequent Southern hybridization to determine the sequence composition of the different PCR fragments. We amplified circular HHV-6 DNA from HSB-2 cells as well as from one of the ciHHV-6 PBMCs (P4) infected with *C. trachomatis* (Figure 5B). In addition, PCR amplified DNA bands were gel purified, cloned and sequenced. Sequencing of PCR products confirmed the results of Southern analysis. The results clearly differentiated three distinct groups of DRs within these samples. We identified a fully reconstituted DR ∼9.7 kb in one fraction of total DNA from HSB-2 cells. Two other fractions contained a ∼8 kb smaller DR and one distinct fragment of ∼3.2 kb was detected in all the fractions of viral DNA from HSB-2 cells as well as in the total genomic DNA isolated after *Chlamydia* mediated reactivation of ciHHV-6 PBMC (P4). Interestingly, these fragments represented reconstituted short DRs, which lacked most of the DR (DR1–DR7) (Figure S5A). We also observed smaller incomplete DRs in HSB-2 cells that were not detected with probes located between DR_L_-T1 and DR_L_-T2 or the telomeric probe (Figures S5B, S5C, S6) indicating recombination between DR_L_-T2 and DR_R_-T1 facilitated by short telomeric repeats. Sequencing reads of variable sizes of HHV-6 DNA containing a single DR confirmed two possible combinations for t-circle formation, either between DR_L_-T2 and DR_R_-T1 or between DR_L_-T2 and DR_R_-T2 resulting in reconstituted DRs. We observed strong variation in the size of the reconstituted DR-T1 (Figure S6). Interestingly, circular HHV-6 DNA with incomplete DR were observed in ciHHV-6 PBMC (P4) only after *Chlamydia* infection, thereby confirming the viral DNA circularization event during chlamydial reactivation of ciHHV-6. Even though circular DNA was present in *Chlamydia* reactivated HSB-ML cells (Figure 3C), we could not detect any circular viral DNA by inverse PCR in these cells. This may be due to sequence variations in the primer-binding region or formation of a longer DR, which cannot be amplified in these PCR conditions.

### Homologous recombination mediated telomere shortening is sufficient for the formation of extra-chromosomal circular HHV-6 molecules

**Figure 6:**
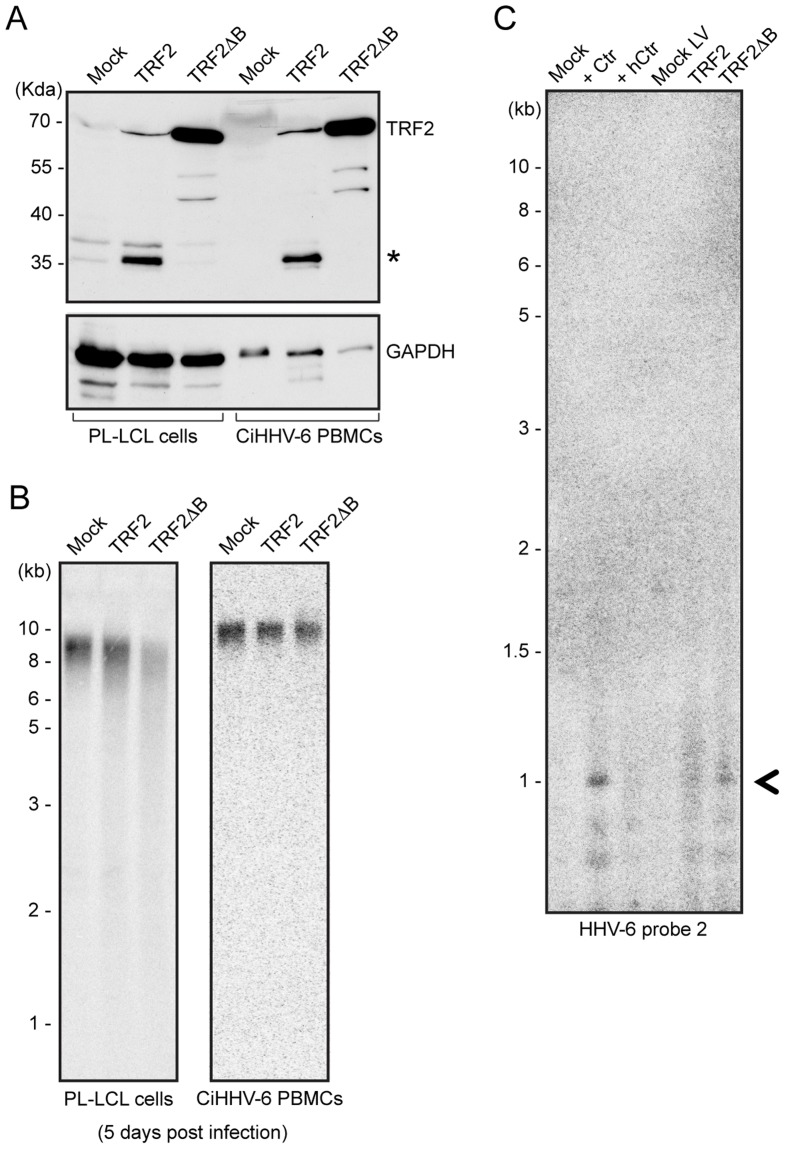
TRF2ΔB overexpression induces formation of circular HHV-6 genome in ciHHV-6 cells. (**A**) TRF2 and TRF2ΔB was over-expressed in an EBV immortalized cell line (PL-LCL) derived from blood cells of a ciHHV-6 person and in freshly isolated PBMCs of another ciHHV-6 person using lentiviral vectors. Protein expression was checked after 3 days of lentivirus infection by Western blot analysis. GAPDH served as loading control. *, Uncharacterized protein. (**B**) Telomere restriction fragment assay was performed in the same cells after 5 days of lentiviral infection using telomere specific probe. (**C**) Formation of circular extra-chromosomal HHV-6 DNA was detected in the same cells by inverse PCR. Data represents the PCR amplifications from PL-LCL cells. As a control PL-LCL cells were infected with *C. trachomatis* or heat inactivated Ctr (hCtr). Amplified PCR product, which was validated by sequencing, is marked with a black arrowhead.

Mammalian telomeric TTAGGG repeats bind the key dimeric DNA binding protein telomere repeat binding factor-2 (TRF2), which plays a key role in maintaining telomere integrity [30]. A mutant of TRF2 with a deletion in the N-terminal basic domain (TRF2ΔB) has previously been shown to induce homologous recombination mediated t-loop formation and subsequent telomere loss [25]. To find experimental evidence for t-circle dependent ciHHV-6 reactivation, we over-expressed human TRF2ΔB (Figure 6A) in various cell types. As expected, TRF2ΔB over-expression induced telomere shortening in all the ciHHV-6 cell types tested (Figure 6B) and caused cell death within 5–7 days after lentivirus infection. Since TRF2ΔB is known to induce t-circle formation, we predicted that circularization of ciHHV-6 would be observed in these cells leading to circular extra-chromosomal viral DNA formation. Extra-chromosomal circular viral DNA with a single DR was detected by inverse PCR (as described in Figure 5) and confirmed by Southern analysis using a HHV-6 specific probe (Figure 6C). Control cells that did not undergo t-circle formation did not contain circular viral DNA molecules. In addition, fragments were purified, cloned and sequenced to verify the origin of the DNA. Thus, our results confirmed the involvement of the host cell t-circle formation machinery in ciHHV-6 reactivation.

## Discussion

Although reactivation of latent HHV-6 has implications in the progression of many diseases including MS and CFS [8], [10], [31], the exact mechanism for latent HHV-6 reactivation, including that of ciHHV-6, remains unknown. We recently described *Chlamydia* infection as a natural trigger to excise the ciHHV-6 genome from the host cell telomere. In this study, we have followed an experimental approach to understand the mechanism of ciHHV-6 reactivation.

We found chromosomal integration of HHV-6A in HSB-2 cells during productive viral infection (Figure S2B). Furthermore, we have previously reported the presence of ciHHV-6 in HeLa cells after HHV-6A infection [32] and other human cell lines have been generated carrying ciHHV-6 [2], [16]. Bearing in mind that about 1% of humans carry genetically inherited HHV-6, we propose that the integration of the HHV-6 genome into human chromosomes is a frequent event. Reactivation of these integrated HHV-6 sequences likely involves their excision from human chromosomes leading to the formation of extra-chromosomal HHV-6 genomes and subsequent replication. HHV-6 contains a potential origin of lytic replication site (OriLyt) within its linear viral genome [33]. However, a linear genome cannot function as a template for viral replication. Therefore, circularization of the genome is required for its continued replication. Although some evidence exists for head-to-tail fusion and circularization of HHV-6 during viral DNA replication [13], [34], this cannot explain the reactivation mechanism of ciHHV-6. For example, head-to-tail end fused viral DNA would maintain the same viral genome size during productive infection with identical lengths of telomeric repeats (DR-T2) within the viral genome. However, we observed viral DNA with varying lengths of telomeric repeats during viral replication (Figures 3A, S5, S6) corroborating similar results published from other laboratories [16], [35], [36]. As previously demonstrated [16], [37], we detected viral DNA with a single reconstituted DR (Figure 5), a DNA configuration that cannot originate from end fusion. Therefore, recombination events involving telomeric repeats within viral genomes are likely necessary for viral DNA circularization and subsequent replication. As HHV-6 integrates within the telomeric repeats located at the ends of human chromosomes, telomeric recombination events may facilitate the excision and circularization of integrated HHV-6.

Since *C. trachomatis* infection reactivates ciHHV-6 replication [17], we investigated the effect of chlamydial infection on host cell telomere integrity. Cells infected with *C. trachomatis* experienced drastic telomere shortening (Figures 2A, 2B, S1A) and subsequent repair, which was dependent on the presence of viable and active bacteria, leading to increased t-circle formation. Changes in telomere length are frequently correlated with increased t-circle formation [25], [26], [38], supporting the hypothesis that *Chlamydia*-mediated telomere alteration initiates circularization of ciHHV-6. In line with this notion, single-stranded viral DNA was detected in *Chlamydia*-infected ciHHV-6 cells (Figure S2C) which is also consistent with the model since telomeric circle formation frequently leads to formation of single-stranded circular telomeric DNA [24]. In addition, we recapitulated ciHHV-6 circularization and viral genome reconstitution by t-circle formation and telomere shortening independent of *Chlamydia* infection by modulating telomeric protein complexes (Figure 6C). Telomeres are regulated and maintained by the multiprotein shelterin complex, which includes TRF2 [18]. TRF2 plays a crucial role in preventing non-homologous end joining at the end of functional telomeres through the formation of t-loops, thereby protecting telomeres from potentially harmful deletions [18]. However, the deletion of the N-terminal basic domain of TRF2 (TRF2ΔB) enhances t-loop formation through t-loop homologous recombination [25]. We utilized these properties of TRF2ΔB to induce t-loop formation in ciHHV-6 cells and showed that enhanced t-loop formation led to weak but definite circularization of viral DNA (Figure 6C). Our results thus provide direct evidence for the involvement of the telomere maintenance machinery in viral reactivation.

It is a well-known phenomenon that HHV-6A and -6B produce high amounts of viral DNA during productive infection but the formation of infectious viral particles is inefficient [39]. We propose that the reactivation of ciHHV-6 from human telomeres is an incomplete process because of the high frequency of shorter t-circle formation that only rarely results in the successful reconstitution of a complete viral genome. Frequent loss of viral DNA between DR1–DR6 in laboratory strains of HHV-6 and packaging of incomplete HHV-6 DNA lacking parts of DR (DR1–DR6) has been previously reported [39]. We have also detected loss of either most of the DR (from DR1–DR8) or parts of the DR (between DR1–DR7) in various cell types during ciHHV-6 reactivation (Figures S5, S6). On the basis of these results, we propose that the infectious nature of HHV-6 genome may be independent of the completeness of the reconstituted circular viral genome corroborating with earlier reports showing the presence of incomplete DR in infectious viral particles [39]. Previous observations of the loss of identical lengths of DR from both ends of HHV-6 DNA [39] supports our model of HHV-6 replication from a circular DNA intermediate containing a single DR. Our results indicate a predominant role of DR_L_-T2 during viral circularization (Figures S5, S6). This reinforces the idea that most of the left part of the viral DR_L_ between pac2 and DR_L_-T2 is preferentially removed from the integrated viral genome thereby producing overhanging viral DNA ends at DR_L_-T2 (Step 3, Figure 1), which can subsequently recombine with the telomeric repeats of DR_R_ (both DR_R_-T1 or DR_R_-T2) to form a reconstituted circular viral genome. The observation of DR_L_-T2 overhangs at the end of chromosome with frequent short telomeric repeats (Figures 4B, 4C) is in line with this hypothesis. Recent studies have utilized single telomere length analysis (STELA) assays to show similar short, unstable telomeric repeats at the sites of HHV-6 integration [16], [40]. However, results obtained with STELA assays should be interpreted with caution since extra-chromosomal telomeric circle-encoded linear DNA containing parts of the viral DR may also be amplified by this technique. To our knowledge, this is the first report to show how the telomere maintenance machinery is exploited to reactivate a latent virus after a prolonged non-infectious state. The enormous prevalence of HHV-6 infection and the possibility of chromosomal integration of other common viruses such as HHV-7, suggests that our data can form a basis to understand HHV-6 reactivation and the subsequent medical consequences for several million people worldwide.

## Materials and Methods

### Blood cells, cell lines, patients and samples

For the study of latent ciHHV-6 activation, established ciHHV-6 cell line, HSB-ML (a tetraploid T-cell line derived from HSB-2 cells with 2–5 copies of ciHHV-6), JL-LCL and PL-LCL were kindly provided by the HHV-6 Foundation, USA (www.hhv-6foundation.org/, www.bioworldantibodies.com). Haploid chronic myeloid cell line KBM-7 [29] was a kind gift from Prof. Thijn R. Brummelkamp. Fresh blood samples from 5 individuals with ciHHV-6 were provided by the HHV-6 Foundation, USA. Viral load was re-verified by qPCR, which confirmed the ciHHV-6 status of these cells. Wild type HeLa (ATCC CCL-2), HeLa229 (ATCC CCL-2.1) and HSB-2 [32] were grown in RPMI 1640 media and 5% fetal bovine serum (FBS) at 37°C and 5% CO_2_. Fresh PBMCs were isolated as described previously [17].

### Ethics statement

The ciHHV-6 blood samples were collected under written informed consent under IRB# CI001-HHV-6 approved by The Essex Institutional Review Board Committee, USA.

### *Chlamydia trachomatis* infection

Cells were infected with *C. trachomatis* at a multiplicity of infection (MOI) of 1–5 as described previously [32].

### DNA extraction

Total DNA was extracted from whole blood samples using QIAamp DNA Blood Mini Kit (Qiagen, Germany) following the manufacturer's protocol. DNA extraction from all the cultured cells was done using DNAzol (Invitrogen) following manufacturer's protocol. In particular, all the experiments involving HHV-6 DNA analysis were carried out using DNA samples extracted with DNAzol as this method is non-invasive and maintain the genomic DNA in a non-shearing form.

### Activation of HHV-6

To study HHV-6 reactivation, ciHHV-6 cell lines and fresh blood samples from individuals with ciHHV-6, were infected with *C. trachomatis* serovar L2 at an MOI of 1–5. Chlamydial infection was monitored by phase contrast microscopy. After 2–3 days of infection, cells were grown in fresh media supplemented with 1 µg/ml of doxycycline, which allowed *Chlamydia*-infected cells to recover.

### Telomere restriction fragment (TRF) assay

For telomere length analysis, total DNA from HeLa229 cells was digested with MspI and HhaI, which do not cut telomeric DNA, for overnight and then separated on a 0.8% agarose gel. These gels were subsequently used for Southern hybridization. As a control, the same DNA samples were digested with HindIII and processed similarly.

### Southern hybridization

For Southern hybridization, agarose gels were incubated in 0.125M HCl for 8–10 min and then in DNA denaturation buffer (1.5M NaCl, 0.5M NaOH) for 30 min. DNA was transferred to Nylon-XL membrane (Amersham Hybond-XL, GE life sciences) by capillary transfer using denaturation buffer for transfer. After transfer, membrane was washed with neutralization buffer (3M NaCl, 0.3M Tri-sodium citrate, 0.5M Tris, pH 8.0) for 15 min and was subsequently pre-incubated in hybridization buffer (GE life sciences, USA). After 1 h of pre-incubation, either random primed probes (GE life sciences, USA) or end labeled probes (Table S1) were added to the hybridization buffer and incubated overnight at 42°C. Membranes were washed and exposed overnight to phosphor storage screens (Fujifilm), which were then scanned by Typhoon 9200 imager (GE Healthcare). PCR amplified Ctr LcrH/SycD gene product of 136 bp was used for random priming and subsequent as probe. For HHV-6A probe 5 and 6 (Figure 4C), PCR products were amplified using primer pairs described in Table S1 and were used for random priming.

### Neutral–neutral 2D DNA electrophoresis

Separation of DNA in neutral–neutral 2D gels was performed as previously described [24]. Briefly, 8–10 µg of DNA was digested with appropriate enzymes and was first separated on 0.4% agarose at low voltage in 0.5× TBE, and the gel was stained with 0.3 µg/ml ethidium bromide. The lane was cut and placed on a clean gel support at 90° to the direction of the first electrophoresis, cast with 1.1% agarose containing 0.3 µg/ml ethidium bromide and run in 0.5× TBE. The first dimension was run overnight at 1 V/cm, and the second at 4 V/cm for 4 h, both at room temperature. Southern blot analyses were performed as described above.

### Extra-chromosomal HHV-6 DNA purification

Total DNA containing HHV-6 DNA was separated using a 0.6% low melting agarose gel for overnight. After ethidium bromide staining, the desired bands were cut out and eluted using the phenol-chloroform extraction method.

### Spreading of double-stranded DNA for TEM

Viral DNA was purified from low melting agarose gel. Prolonged exposure to UV light was prevented in order to avoid any DNA break. For visualizing double-stranded DNA, purified DNA in TE-buffer was mixed with ammonium acetate and cytochrome c (50–200 ng DNA in 2–10 µl TE-buffer, 200 µl 0.2 M ammonium acetate, 0.8–2.0 µl of 1% cytochrome c [in distilled water]) and 100 µl drops were placed on parafilm. After incubation for 20 min at room temperature, the cytochrome c coated DNA was picked up by touching collodion-coated grids to the surface of the drops. Grids were immediately stained with 5×10^−5^ M uranyl acetate in 90% ethanol or 30 sec, washed for 30 sec in 90% ethanol, air dried and metal coated with platinum/palladium by rotary shadowing under an angle of 5–7%.

### Inverse PCR to detect short t-circles

100 ng of total genomic DNA was used to amplify short t-circles using a primer pair facing against each other (see table S1) and Phusion high-fidelity master mix with GC buffer (Thermo scientific). The following amplification cycles were used: Initial denaturation at 98°C for 2 minutes, 28 cycles of denaturation at 98°C for 30 seconds, primer annealing at 64°C for 30 seconds and primer extension at 72°C for 7 minutes. Final extension was done at 72°C for 30 minutes. Amplified PCR products were run on 1% agarose gel and were used for Southern hybridization. Amplified PCR products were cloned into TOPO 2.1 vector and sequenced using M13 forward and reverse primers.

### Inverse PCR for the detection of circular HHV-6 genome with single DR

100 ng of total genomic DNA was used to amplify circular or concatemeric HHV-6 DNA having a single direct repeat (DR) using a primer pair facing against each other (see table S1) and LA Taq (Takara Biosciences). Long PCR amplification was performed as follows: an initial cycle of denaturation at 92°C for 4 minutes was followed by 10 cycles of denaturation at 92°C for 10 seconds, primer annealing at 64°C for 30 seconds and primer extension at 68°C for 6 min, followed by 20 additional cycles under similar conditions except that the primer extension time was increased for 20 seconds per subsequent cycle. PCR was terminated with a final extension step for 30 minutes at 72°C. Amplified PCR products were run on 1% agarose gels and subjected to Southern hybridization. Amplified PCR products were cloned into TOPO 2.1 vector and sequenced using M13 forward and reverse primers. For inverse PCR in Figure 6C, a different primer pair (For3 and Rev3, see Table S1) was used.

### FISH

FISH and Co-FISH experiments were performed using the following protocol. Metaphase spreads were prepared after 2–3 hrs of colcemid treatment using standard cell biology techniques. For co-FISH, slides were hybridized with 2 different probes using previously described techniques [41]. For co-FISH with blood cells, PBMCs were stimulated for 72 h with 10 µg/mL PHA and then cultured in RPMI1640 media containing 100 units/mL IL-2 and 10% FCS. A custom designed Alexa-488 tagged PNA oligo probe (Panagene, South Korea) against HHV-6 (Alexa488-OO-GCG TCA TAA TGC TCA ACA-CONH_2_) was used for FISH analysis using manufacturer's protocol. Alexa488-tagged Tel-G probes and Cy5-tagged Tel-C probe were purchased from Eurogentec, Germany (Cat No. PN-TC055-005, TG055-005). For single chromatid telomere staining (Figure 2D), HeLa cells were incubated with 5-bromo-2′-deoxyuridine (BrdU) and 5-bromo-2′-deoxycytidine (BrdC). Newly synthesized DNA strands were subsequently digested with Exonuclease III.

### Sequence analysis of extra-chromosomal HHV-6 DNA

To validate the origin of extra-chromosomal HHV-6 DNA from the ciHHV-6, we separated extra-chromosomal HHV-6 DNA from the chromosomal DNA by agarose gel electrophoresis. DNA from both the fractions were eluted and used for amplification of viral U94 ORF by PCR. Amplified DNA was cloned into TOPO 2.1 vector and sequenced.

### Lentivirus preparation and overexpression of TRF2ΔB

Constructs for TRF2 and TRF2ΔB overexpression [25] were obtained from Addgene, USA. Detailed protocol for lentivirus generation and infection are previous described [42]. Rabbit monoclonal TRF2 antibody was purchased from Abcam, UK (Cat no. ab108997).

## Supporting Information

Figure S1 Alteration of telomere length during *Chlamydia* infection. (A) *Chlamydia trachomatis* (Ctr) infection induces telomere shortening without causing genomic DNA degradation. HeLa229 cells were infected with *C. trachomatis* for different time intervals. To one of the samples, doxycycline (1 µg/ml) (Dox) was added after 24 h of *Chlamydia* infection and allowed to grow for another 48 h. Only doxycycline treated HeLa229 cells are used as control. Total genomic DNA (10 µg) was digested either with MspI and HhaI or with HindIII and separated by agarose gel electrophoresis. Telomeric sequences were detected by Southern hybridization using an end-labeled (CCCTAA)_4_ probe (Tel-C probe). The position of molecular weight markers run is shown on the left. An end-labeled probe against genomic GAPDH sequence (see table S1) was used to check DNA quality and was visualized using autoradiography. The gel was subsequently stripped and hybridized with an end-labeled probe against chlamydial DNA (Ctr probe). Mock, Cells with no *Chlamydia* infection. (B) Schematic diagram showing running pattern of different forms of DNA in neutral-neutral 2D DNA electrophoresis. ssG DNA, single stranded G-rich telomeric DNA. (C) Detection of circular telomeric DNA in HeLa cells after *Chlamydia* infection. HeLa cells were either mock infected or with *Chlamydia trachomatis* (Ctr) for 24 h. Subsequently, infected cells were cultured for 2 more days in presence of 1 µg/ml of doxycycline. 8 µg of total genomic DNA from each sample was processed for 2D DNA electrophoresis and subsequent Southern hybridization using Tel-C probe. Black arrowhead indicates circular telomeric DNA. Mock, without *Chlamydia* infection. dpi, days post infection.
(TIF)

Figure S2 (A) HSB-2 cells infected with HHV-6A were studied for infectious viral particle formation by transmission electron microscopy. Numerous infectious viral particles are observed in most of the cells. (B) FISH analysis was used to study chromosomal integration of HHV-6A in the same HSB-2 cells. A custom designed Alexa488-tagged PNA oligonucleotide probe against HHV-6 DNA was used to detect integrated HHV-6 DNA. Co-hybridization with Cy5-tagged Tel-C probe was used to study telomeric co-localization. HHV-6A integrated chromosome is highlighted. (C) 8 µg of total genomic DNA from HSB-2 cells infected with HHV-6A and 16 µg of total genomic DNA from HSB-ML cells infected with *C. trachomatis* (Ctr) were digested with MspI and HhaI and were processed for 2D-DNA electrophoresis and subsequent Southern hybridization with telomere specific probes. (D) 8 µg of total genomic DNA from HSB-2 cells without having HHV-6A infection and HSB-ML cells without *Chlamydia* infection (control) were digested with MspI and HhaI and were processed as mentioned above. (E) Samples described under (B) were digested with S1 nuclease and similarly processed as described above.
(TIF)

Figure S3 Detection of short t-circles originating from HHV-6 DR_L_ by Southern hybridization. (A) Total genomic DNA from HSB-2 cells having ciHHV-6 as well as productive HHV-6 infection was digested with either BamHI or HindIII and run on a 1% agarose gel and processed for Southern hybridization. The membrane was probed first with HHV-6 probe 2, stripped and re-probed with HHV-6 probe 1. Finally the membrane was probed with telomeric-C probe. Desired bands, which are detected by both the probes, are marked with blue arrow. HSB-2 total DNA without having viral infection served as a negative control. Bands detected by both the HHV-6 probes are indicated with blue arrowhead. (B, C) Diagrammatic representation of possible band sizes after BamHI and HindIII digestion of HSB-2 DNA having ciHHV-6 as well as productive HHV-6 infection. Approximate location of HindIII or BamHI digestion sites within and around viral DR are marked with dotted lines. The location of probes used to detect specific regions of viral DNA and their expected sizes are indicated. Positions of restriction digestion sites and expected band lengths are based on HHV-6A (U1102) genome (X83413.1).
(TIF)

Figure S4 (A) KBM-7 cells infected with HHV-6A were studied for infectious viral particle formation by transmission electron microscopy. Numerous viral nuclear capsids (marked with white arrowhead) were observed in most of the cells. (B) Diagram of the principle of inverse PCR to detect short t-circles. The position of the forward (For1) and reverse (Rev1) primers is indicated (see table S1 for primer sequence information).
(TIF)

Figure S5 Sequence details of circular/concatemeric HHV-6 DNA having single incomplete DR. (A) Sequence details of ∼3.2 kb band from Figure 5B showing reconstitution of incomplete DR by fusion of DR_L_ with a part of DR_R_. (B, C) Sequence details of reconstituted incomplete DR formed by fusion of DR_R_-T1 and DR_L_-T2. Full-length as well as short DR sequences were PCR amplified using a pair of primer facing against each other as described in Figure 5. Amplified sequences are cloned and sequenced. Short DRs originating from telomeric repeats confirm telomere mediated DR reconstitution.
(TIF)

Figure S6 Strong sequence variation in HHV-6 DR-T1 as revealed by sequencing of circular/concatemeric HHV-6 DNA having single incomplete DR. (A–C) Sequence details of reconstituted incomplete DR formed by fusion of DR_R_-T1 and DR_L_-T2. Variable length of telomeric repeats at the junction site is marked with red font. Full-length as well as short DR sequences are PCR amplified using a pair of primer facing against each other as described in Figure 5. Amplified sequences are cloned into TOPO 2.1 vector and sequenced. Short DRs originating from telomeric repeats confirm telomere mediated DR reconstitution.
(TIF)

Table S1 Oligos used for PCR and Southern hybridizations.
(PDF)

Text S1 Sequence information of inverse PCR showing junction regions within short t-circles formed from viral DR. (A) Sequence details of the junction region as derived from approximately 1.7 kb short t-circle PCR products isolated from HSB-2 cell DNA with HHV-6A infection (see Figure 4D). (B) Five different length short t-circle PCR products (see Figure 4D), more than 2 kb in size and isolated from HSB-ML cells, shows similar sequence homology with variable size of telomeric repeats.
(PDF)
