# Supplementary figures and images for: Reactivation of Chromosomally Integrated Human Herpesvirus-6 by Telomeric Circle Formation
Source: PLoS Genet. 2013 Dec 19;9(12):e1004033. doi: 10.1371/journal.pgen.1004033 (PMC3868596; doi:10.1371/journal.pgen.1004033)

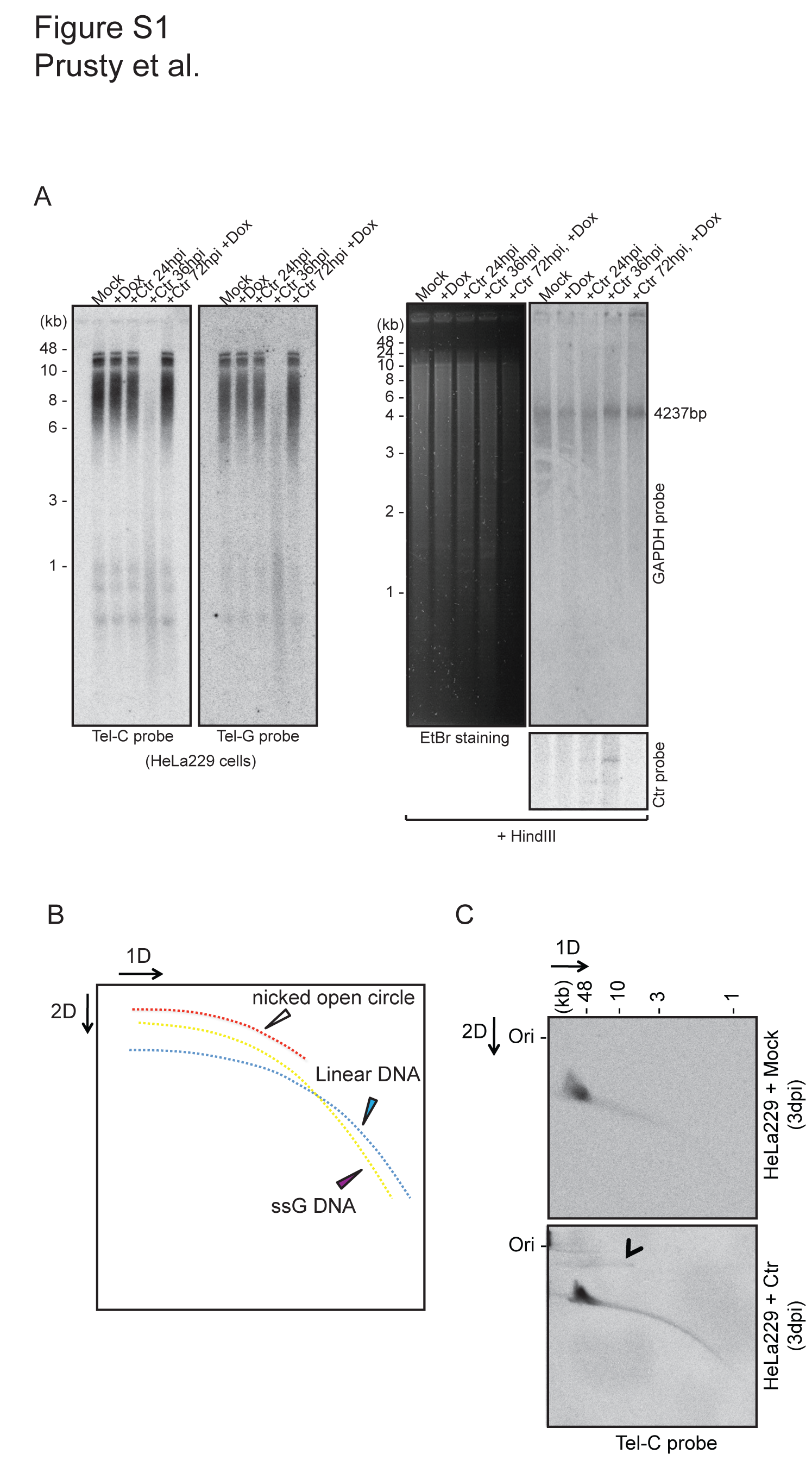

Supplement: Figure S1 — Alteration of telomere length during Chlamydia infection. (A) Chlamydia trachomatis (Ctr) infection induces telomere shortening without causing genomic DNA degradation. HeLa229 cells were infected with C. trachomatis for different time intervals. To one of the samples, doxycycline (1 µg/ml) (Dox) was added after 24 h of Chlamydia infection and allowed to grow for another 48 h. Only doxycycline treated HeLa229 cells are used as control. Total genomic DNA (10 µg) was digested either with MspI and HhaI or with HindIII and separated by agarose gel electrophoresis. Telomeric sequences were detected by Southern hybridization using an end-labeled (CCCTAA)4 probe (Tel-C probe). The position of molecular weight markers run is shown on the left. An end-labeled probe against genomic GAPDH sequence (see table S1) was used to check DNA quality and was visualized using autoradiography. The gel was subsequently stripped and hybridized with an end-labeled probe against chlamydial DNA (Ctr probe). Mock, Cells with no Chlamydia infection. (B) Schematic diagram showing running pattern of different forms of DNA in neutral-neutral 2D DNA electrophoresis. ssG DNA, single stranded G-rich telomeric DNA. (C) Detection of circular telomeric DNA in HeLa cells after Chlamydia infection. HeLa cells were either mock infected or with Chlamydia trachomatis (Ctr) for 24 h. Subsequently, infected cells were cultured for 2 more days in presence of 1 µg/ml of doxycycline. 8 µg of total genomic DNA from each sample was processed for 2D DNA electrophoresis and subsequent Southern hybridization using Tel-C probe. Black arrowhead indicates circular telomeric DNA. Mock, without Chlamydia infection. dpi, days post infection. (TIF) [file pgen.1004033.s001.tif]

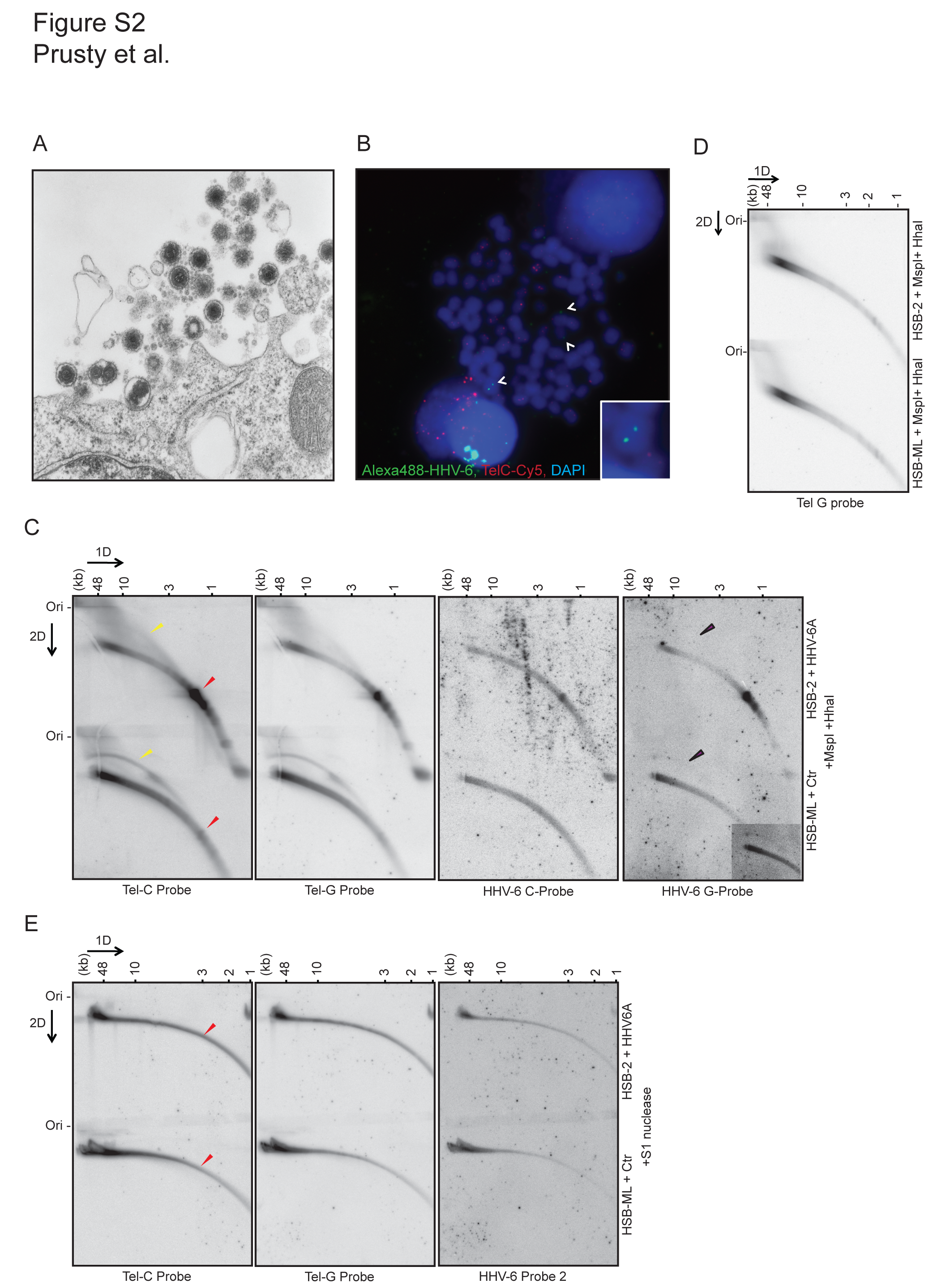

Supplement: Figure S2 — (A) HSB-2 cells infected with HHV-6A were studied for infectious viral particle formation by transmission electron microscopy. Numerous infectious viral particles are observed in most of the cells. (B) FISH analysis was used to study chromosomal integration of HHV-6A in the same HSB-2 cells. A custom designed Alexa488-tagged PNA oligonucleotide probe against HHV-6 DNA was used to detect integrated HHV-6 DNA. Co-hybridization with Cy5-tagged Tel-C probe was used to study telomeric co-localization. HHV-6A integrated chromosome is highlighted. (C) 8 µg of total genomic DNA from HSB-2 cells infected with HHV-6A and 16 µg of total genomic DNA from HSB-ML cells infected with C. trachomatis (Ctr) were digested with MspI and HhaI and were processed for 2D-DNA electrophoresis and subsequent Southern hybridization with telomere specific probes. (D) 8 µg of total genomic DNA from HSB-2 cells without having HHV-6A infection and HSB-ML cells without Chlamydia infection (control) were digested with MspI and HhaI and were processed as mentioned above. (E) Samples described under (B) were digested with S1 nuclease and similarly processed as described above. (TIF) [file pgen.1004033.s002.tif]

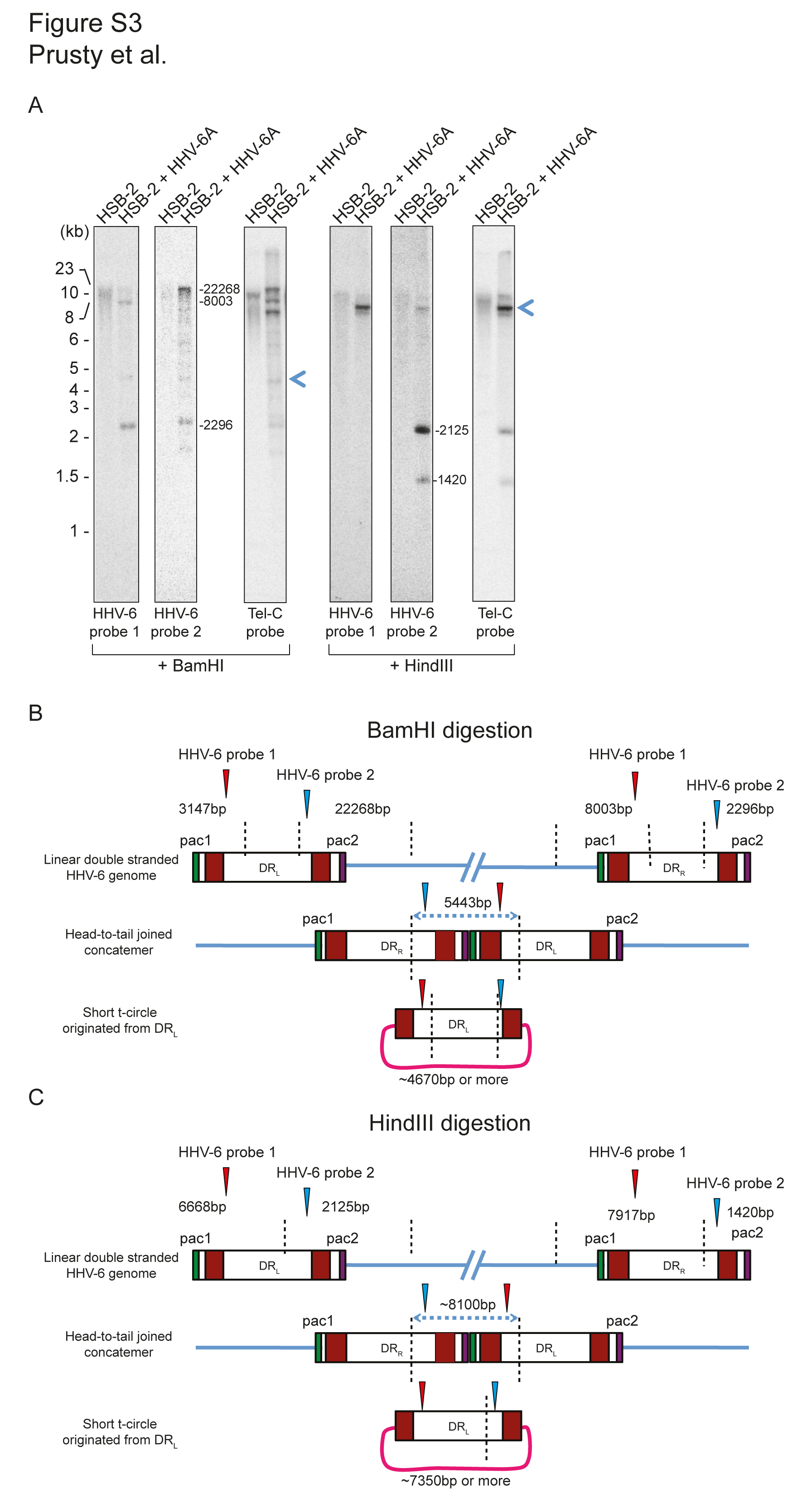

Supplement: Figure S3 — Detection of short t-circles originating from HHV-6 DRL by Southern hybridization. (A) Total genomic DNA from HSB-2 cells having ciHHV-6 as well as productive HHV-6 infection was digested with either BamHI or HindIII and run on a 1% agarose gel and processed for Southern hybridization. The membrane was probed first with HHV-6 probe 2, stripped and re-probed with HHV-6 probe 1. Finally the membrane was probed with telomeric-C probe. Desired bands, which are detected by both the probes, are marked with blue arrow. HSB-2 total DNA without having viral infection served as a negative control. Bands detected by both the HHV-6 probes are indicated with blue arrowhead. (B, C) Diagrammatic representation of possible band sizes after BamHI and HindIII digestion of HSB-2 DNA having ciHHV-6 as well as productive HHV-6 infection. Approximate location of HindIII or BamHI digestion sites within and around viral DR are marked with dotted lines. The location of probes used to detect specific regions of viral DNA and their expected sizes are indicated. Positions of restriction digestion sites and expected band lengths are based on HHV-6A (U1102) genome (X83413.1). (TIF) [file pgen.1004033.s003.tif]

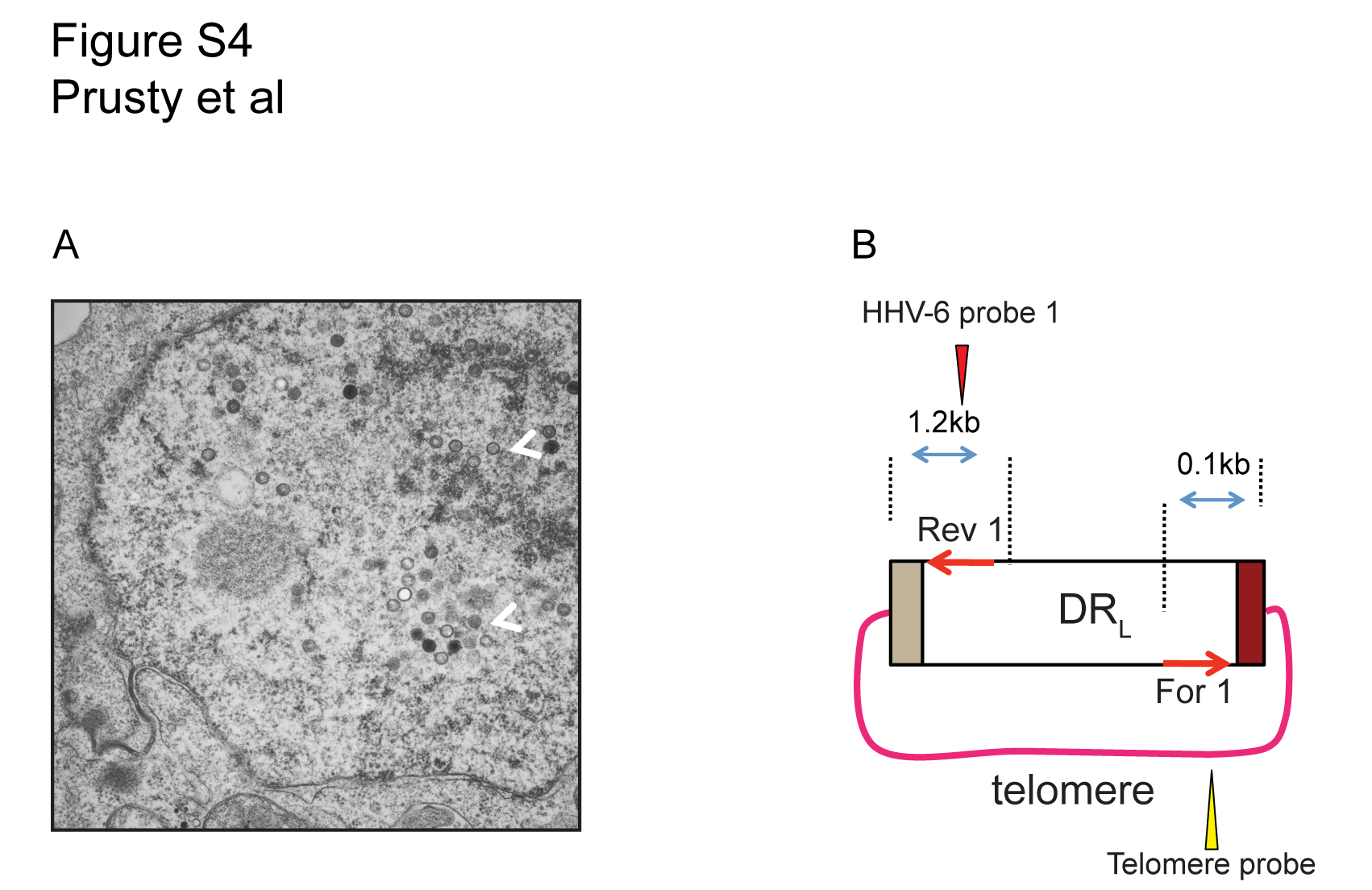

Supplement: Figure S4 — (A) KBM-7 cells infected with HHV-6A were studied for infectious viral particle formation by transmission electron microscopy. Numerous viral nuclear capsids (marked with white arrowhead) were observed in most of the cells. (B) Diagram of the principle of inverse PCR to detect short t-circles. The position of the forward (For1) and reverse (Rev1) primers is indicated (see table S1 for primer sequence information). (TIF) [file pgen.1004033.s004.tif]

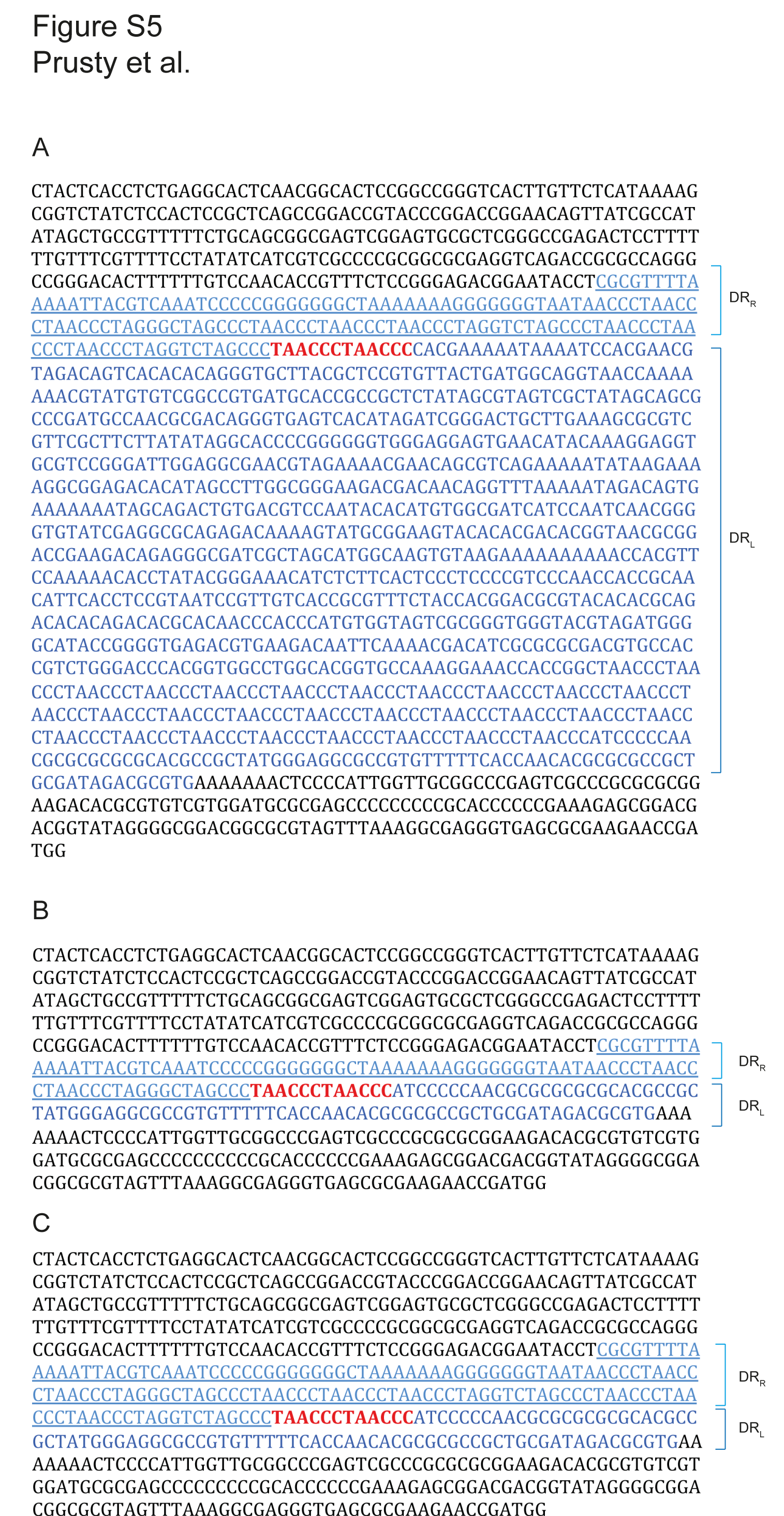

Supplement: Figure S5 — Sequence details of circular/concatemeric HHV-6 DNA having single incomplete DR. (A) Sequence details of ∼3.2 kb band from Figure 5B showing reconstitution of incomplete DR by fusion of DRL with a part of DRR. (B, C) Sequence details of reconstituted incomplete DR formed by fusion of DRR-T1 and DRL-T2. Full-length as well as short DR sequences were PCR amplified using a pair of primer facing against each other as described in Figure 5. Amplified sequences are cloned and sequenced. Short DRs originating from telomeric repeats confirm telomere mediated DR reconstitution. (TIF) [file pgen.1004033.s005.tif]

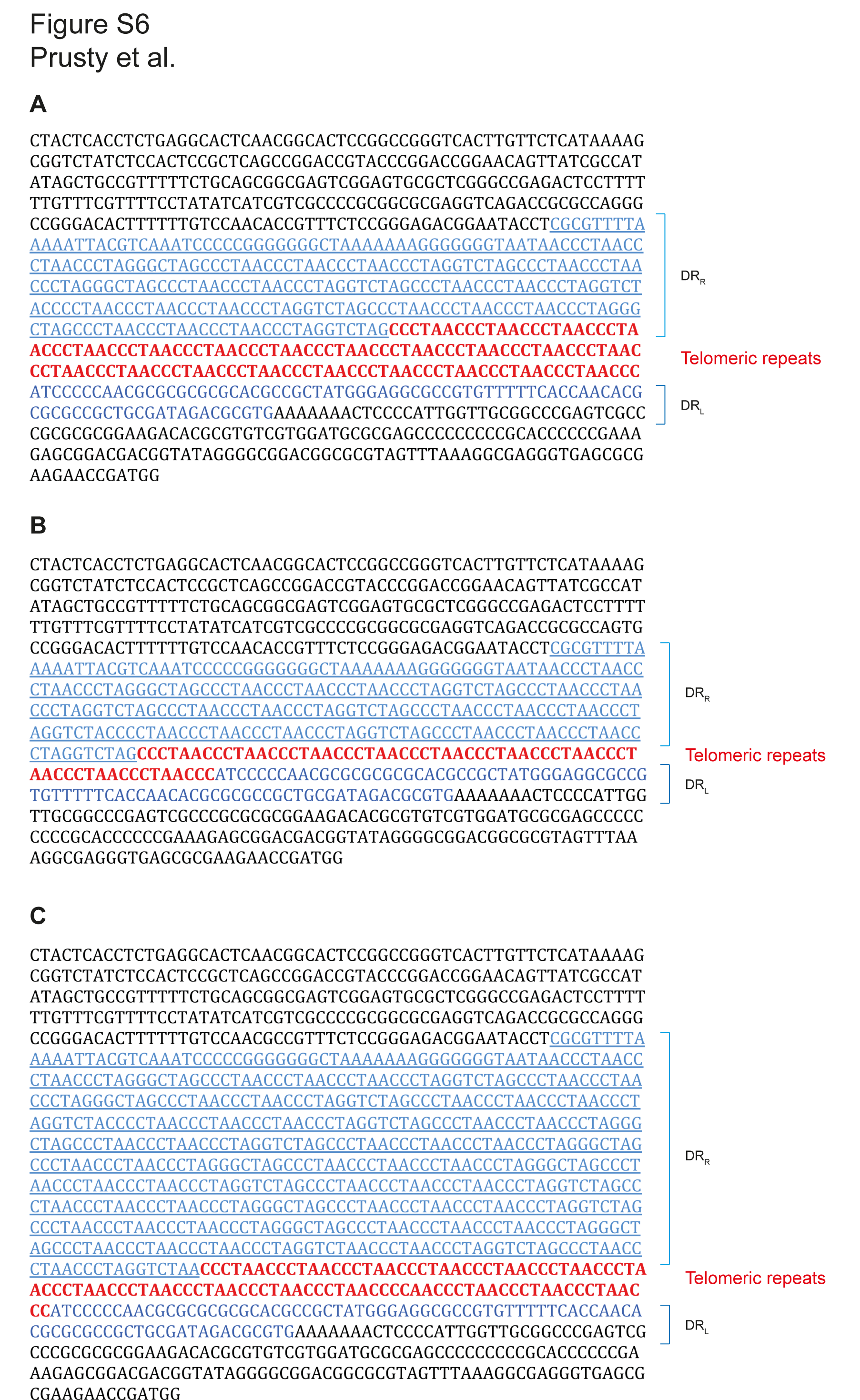

Supplement: Figure S6 — Strong sequence variation in HHV-6 DR-T1 as revealed by sequencing of circular/concatemeric HHV-6 DNA having single incomplete DR. (A–C) Sequence details of reconstituted incomplete DR formed by fusion of DRR-T1 and DRL-T2. Variable length of telomeric repeats at the junction site is marked with red font. Full-length as well as short DR sequences are PCR amplified using a pair of primer facing against each other as described in Figure 5. Amplified sequences are cloned into TOPO 2.1 vector and sequenced. Short DRs originating from telomeric repeats confirm telomere mediated DR reconstitution. (TIF) [file pgen.1004033.s006.tif]
