# Supplementary material for: Reactivation of Chromosomally Integrated Human Herpesvirus-6 by Telomeric Circle Formation
Source: PLoS Genet. 2013 Dec 19;9(12):e1004033. doi: 10.1371/journal.pgen.1004033 (PMC3868596; doi:10.1371/journal.pgen.1004033)
Supplement: Table S1 — Oligos used for PCR and Southern hybridizations. (PDF) [file pgen.1004033.s007.pdf]

Table S1: Oligo sequences used for southern hybridization, PCR.

Gene	Sequence (5' →3')	
**Oligos for Southern Hybridization**		
HHV-6 Probe 1	ACGTGCTGCAAGGCCGACTC	
HHV-6 Probe 2<br>(HHV-6 G-probe)	CTTACACTTGCCATGCTAGC	
HHV-6 Probe 3	GCGTGTGTGTTTTCGCTCA	
HHV-6 Probe 4	GTGTGATAAGTGCTGCAACG	
Tel-G probe	TTAGGGTTAGGGTTAGGGTTAGGG	
Tel-C probe	CCCTAACCCTAACCCTAACCCTAA	
Ctr probe	AGGCGTACGATTTGATCGCCAA	
GAPDH probe	GCAACTTGGCAAATCAAAGCCCTGGGACTAGGGGG	
HHV-6 C-probe	GCTAGCATGGCAAGTGTAAG	
**Primers used for short t-circle inverse PCR**		
For1	CGTGTGTACGCGTCCGTGGTAG	
Rev1	CGCAGCAGCTCCAGGTCGTC	
**Primers used to amplify HHV-6 circular DNA with single DR**		
For2	ATGATTTCCGATAGCGTCCG	
Rev2	GCGTGTGTGTTTTCGCTCA	
For3	CTACTCACCTCTGAGGCACT	
Rev3	CATCGGTTCTTCGCGCTCAC	
**Primers used to generate HHV-6A probe 5**		
For	AGTTTAAAGGCGAGGGTGAGC	
Rev	TCCTCGGGAACGACCGC	

**Primers used to generate HHV-6A probe 6**		
For	GCACAACCCACCCATGTGGTAGTCGCGG	
Rev	GTGGTTTCCTTTGGCACCGT	
